# Introducing *Reproduction and Fertility*

**DOI:** 10.1530/RAF-20-0001

**Published:** 2020-07-09

**Authors:** Norah Spears, Andrew W Horne

**Affiliations:** 1 University of Edinburgh – Biomedical Sciences, Edinburgh, UK; 2 University of Edinburgh – MRC Centre for Reproductive Health, Edinburgh, UK

When the *Journal of Reproduction and Fertility* (now known as *Reproduction*) was launched 60 years ago in 1960, it addressed the need for a formal publication to represent the members of what was then the Society for the Study of Fertility (now the Society for Reproduction and Fertility; SRF). Through the years, the journal’s successive editorial boards have molded it into one that has the reputation of being a leading title in reproductive biology.

In recent years, the SRF identified the need for a new publication outlet for its members and authors, one which is fully open access and devoted to the entire breadth of our subject, from basic through to translational to clinical research. With this endpoint in mind, SRF, alongside the society’s established publisher Bioscientifica, are launching a new fully open-access journal, *Reproduction and Fertility*, and have invited us to lead the journal as founding co-editors-in-chief.

We have conceived new editorial policies that, together with the publication of all work under open-access licenses, will allow the widest possible dissemination of published work:

1. We have made the decision to focus on scientific robustness when assessing papers, rather than on a perceived level of interest or originality. Too often, editorial boards find themselves needing to cherry pick only what is considered to be the most exciting or novel research for publication, with negative results, confirmatory studies or minor advances often overlooked. Here, our aim is simply to encourage submission of all papers which are scientifically and methodologically sound.
2. Articles can be in the form of research articles, research letters, reviews or commentaries. Our inclusion of short research letters provides authors with an opportunity to let the scientific community know of preliminary work prior to completion of a more comprehensive study or to publish small but scientifically robust studies where that avenue of research has not been further pursued.
3. In alignment with the greater visibility proffered by publishing articles open access, authors will be asked to provide a short Lay Summary of their article, a condensed summary intended for the public or those working outside of the immediate field. These summaries will offer authors the opportunity to share their perspective on the wider impact of their work.
4. Authors will also be encouraged to include a graphical abstract that will sit alongside their traditional text abstract. The graphical abstract is intended to be a neat, visual summary of the key points of the article and will serve as an additional peer-reviewed medium for communicating authors’ work within the journal and using social-media platforms.
5. Finally, it is well known that open-access articles are freely and universally accessible online. Authors hold the copyright for their work and are able to comply with any requirements of funding bodies for work to be published via a gold open-access route. However, open-access publication shifts the onus of payment from subscribing libraries to the author. We are excited to announce that SRF and Bioscientifica will completely cover the article publishing charges for *Reproduction and Fertility* papers during the launch years, to enable authors to benefit from the additional exposure afforded by open-access publication at no extra cost. This is an extraordinary move and one that will support SRF members and the wider scientific community.

Our vision for this new journal is that it will draw from the opportunities presented to create a powerful new outlet to accelerate the research, education and practice of those working in our field and beyond. Scientific and medical breakthroughs are rare, increasingly so; rather, iterative progress is the norm. We need to advocate for openness, visibility, collaborative and holistic working in order to provide an environment in which many iterations can, together, have the same impact as those breakthrough moments, increasing our knowledge of reproduction and fertility and improving reproductive health. We are currently recruiting an exceptional and diverse editorial board with the necessary experience and expertise to shape the path for this new journal and one that reflects the wide scope of basic, translational, and clinical reproduction and fertility.

*Reproduction and Fertility* has been conceived as a complementary journal to *Reproduction* and will benefit from *Reproduction’s* tried-and-tested editorial policies and from its established standards for rigor during peer review. There are many good papers submitted to *Reproduction* which cannot be published there. As such, we will also work alongside the editorial board of *Reproduction* to identify manuscripts that might be considered for *Reproduction and Fertility*, including aiming to use reviewers’ reports, to save authors time and to allow their papers to be published in an SRF journal.

*Reproduction and Fertility* is now accepting submissions of research articles, research letters, reviews and commentaries. We anticipate that *Reproduction and Fertility* will be included in PubMed Central and Journal Citation Reports (Science Citation Index Expanded) as soon as the required number of articles have been published, at which point indexing will be applied to all articles retrospectively.

We hope that you take advantage of the extraordinary offer to publish open-access free of charge and look forward to seeing your submissions.

## Declaration of interest

The authors declare that there is no conflict of interest that could be perceived as prejudicing the impartiality of this commentary.

